# Early-life undernutrition, immune dysregulation, and cancer incidence in later life: a national life-course analysis from the China health and retirement longitudinal study

**DOI:** 10.3389/fimmu.2025.1602290

**Published:** 2025-08-27

**Authors:** Hailin Wang, Changkang Wu, Xuancheng Zhou, Gang Huang, Jingdong Li, Xiaowei Tang

**Affiliations:** ^1^ Department of Hepatobiliary Surgery, Affiliated Hospital of North Sichuan Medical College, Nanchong, China; ^2^ Department of Gastroenterology, The Affiliated Hospital of Southwest Medical University, Luzhou, China

**Keywords:** early-life undernutrition, cancer incidence, childhood famine exposure, nutritional deprivation, immune function, chronic inflammation

## Abstract

**Background:**

Severe childhood famine may imprint durable immunometabolic scars, yet its longitudinal impact on chronic inflammation and cancer trajectories in China’s ageing population is unresolved.

**Methods:**

We analyzed 2–515 adults in the China Health and Retirement Longitudinal Study (2011–2015) who were born 1947–1961; early-life undernutrition was assigned when birth occurred during 1959–1961 and in one of five provinces with > 30% grain deficit. In parallel, an independent hospital-based verification cohort of 82 adults (recruited 2024–2025) underwent identical exposure classification, biomarker sampling, and cancer surveillance for external validation. High-sensitivity C-reactive protein (hs-CRP), white-blood-cell (WBC) counts, and physician-confirmed malignancies were the prespecified outcomes. Multivariable logistic and Cox mixed-effects models, with interaction terms, quantified dose-response relations and effect modification; estimates from both cohorts were pooled with inverse-variance weighting.

**Results:**

Forty-one percent of CHARLS respondents met undernutrition criteria. Compared with unexposed peers, exposed adults showed higher mean hs-CRP (3.18 ± 2.36 *vs* 2.74 ± 2.11 mg L^-^¹) and modestly elevated median WBC (6.6 *vs* 6.3 × 10^9^ L^-^¹). Undernutrition independently increased the odds of chronic inflammation (hs-CRP ≥ 3 mg L^-^¹: OR 1.46, 95% CI 1.22–1.75) and leucocytosis (WBC > 10 × 10^9^ L^-^¹: OR 1.28, 1.04–1.57). Over 9–722 person-years, 122 new cancers occurred; exposed individuals faced a 59% higher hazard (HR 1.59, 1.11–2.27). The verification cohort produced concordant estimates (pooled HR 1.63, 1.23–2.11). Associations were strongest among adults ≥ 60 y or harboring ≥ 2 baseline comorbidities (p-interaction < 0.05).

**Conclusion:**

Developmental caloric deprivation leaves a lasting inflammatory fingerprint that translates into excess mid-life cancer burden. Life-course screening for famine survivors coupled with anti-inflammatory and nutritional interventions will curb malignancy risk as China’s cohort of famine-exposed elders expands.

## 1 Introduction

Early-life undernutrition (a quantitative energy–protein deficit during gestation, infancy or early childhood) is conceptually distinct from childhood famine exposure (an abrupt, population-level collapse of the food system) and from stunting (a phenotypic outcome of chronic linear-growth failure); nonetheless, all three constructs share the common feature of nutritional insufficiency during sensitive developmental windows (1–3).For this analysis we retained the construct early-life undernutrition because it captures both prenatal and early-childhood scarcity, aligns with the Developmental Origins of Health and Disease (DOHaD) paradigm, and can be operationalized in CHARLS via birth-year × province criteria. Similar to frailty and other early-adversity phenotypes, childhood undernutrition reflects diminished physiological reserves and an increased susceptibility to various health stressors (4–6). Early-life undernutrition has been widely identified among middle-aged and older populations who experienced famine or prolonged food insecurity in their childhood (7–9). Previous studies conducted in different countries have reported varying prevalence rates of undernutrition and its sequelae among individuals exposed to famine, with an estimated 5–15% (10–12) prevalence in certain historically affected cohorts. In China, especially among those born in the late 1950s and early 1960s, there is compelling evidence of severe childhood famine exposure (13–15). According to historical data, many of these individuals encountered pronounced nutritional deficits, often leading to higher incidence of chronic illnesses. With the aging of China’s population, early-life undernutrition constitutes one significant concern for healthcare systems, presenting a considerable public health burden (16–18).

Similar to the concept of frailty as a geriatric syndrome, early-life undernutrition may have enduring effects on health outcomes in older age. A range of literature has extensively documented the long-term associations between inadequate childhood nutrition and adverse health consequences, including impaired growth, metabolic disorders, and impaired organ function (19–22). However, less attention has been paid to two critical adult health outcomes: immune dysfunction and incident malignancies. Immune dysfunction can manifest as chronically elevated inflammatory markers and compromised immune surveillance; concurrently, cancer incidence rises with age and may be influenced by an individual’s lifetime exposure to stressors. Collectively, mounting research posits that developmental insults—undernutrition in particular—could set in motion physiological and epigenetic pathways that predispose individuals to an elevated risk of inflammation-related conditions and oncogenesis. Guided by the life-course/DOHaD model, we hypothesize that famine-era nutrient deprivation imprints epigenetic marks such as IGF2 hypomethylation and IL-6 promoter hypermethylation, thereby programming a pro-inflammatory milieu, impairing immune surveillance, and accelerating tumor initiation and promotion.

The immune system fulfills an essential role in shielding the body from various pathogens and in regulating inflammation, and undernutrition during sensitive periods may compromise its maturation. Investigations have indicated that nutritional scarcity in early years could lead to heightened systemic inflammation, partly via lower resilience to stress and suboptimal immune cell functioning (23–25). Simultaneously, it has been proposed that persistent inflammation accelerates cellular damage and fosters tumorigenic processes over time (26, 27). Although a few studies allude to associations between child adversity and higher cancer risk in adulthood, the exact role of early-life undernutrition in shaping immune trajectories and incident malignancies has not been thoroughly explored within a large population-based Chinese cohort. Given the interplay between undernutrition, chronic inflammation, and cancer, further examination of these links is warranted.

Activities of daily living and frailty research have illuminated how early functional deficits can predict major health outcomes and mortality. Drawing parallels, early-life adversity—especially in the form of inadequate nutrition—could represent a catalyst for adult immune dysfunction and cancer onset, with multiple mechanistic pathways converging (28–30). Individuals who experienced severe undernutrition in childhood may develop smaller organ size and reduced cellular reserves, thereby increasing their vulnerability to immunologic insults and neoplastic changes. Chronic illnesses such as cardiovascular disease, diabetes, and other comorbidities may also emerge more frequently in this population, compounding the eventual burden of disease (31–33). However, current research predominantly addresses either immune dysregulation or cancer risk and seldom integrates both outcomes to capture a full profile of late-life health. Consequently, a comprehensive understanding of how childhood undernutrition can affect immune parameters and malignancy incidence in mid to late adulthood remains elusive.

Despite accumulating evidence from observational studies and smaller regional cohorts, few large-scale analyses have systematically evaluated the cumulative effect of early-life undernutrition on adult immune markers and cancer onset, especially in the context of China’s nationwide aging population. To address this knowledge gap, it is crucial to employ a life-course perspective, investigating participants from historically famine-affected regions in their mid and later years, when immune changes and cancer diagnoses are most likely to manifest. Additionally, there is a pressing need to determine whether any associations between undernutrition exposure and health outcomes are moderated by adult social or lifestyle factors, such as education, urban/rural residence, or baseline comorbidity profiles. Understanding these interactions may help contextualize the extent to which childhood nutrition deficits exert a lasting influence on the trajectory of immune and oncologic risks.

In the study, we utilized a four-year (2011 to 2015) longitudinal dataset from the China Health and Retirement Longitudinal Study (CHARLS), a nationally representative sample encompassing individuals aged 45 years and older. This large-scale data source allows for an in-depth exploration of how early-life undernutrition, as defined by both historical famine references and birth-year criteria, impacts immune function indicators and the incidence of cancer in mid to late adulthood. We hypothesized that participants who experienced substantial nutritional deprivation in childhood would exhibit higher levels of inflammatory markers (e.g., CRP, abnormal WBC counts) and a greater incidence of cancer during follow-up, compared to individuals who did not report or were not confirmed to have undernutrition exposure. We further speculated that the cumulative detrimental effects of early-life nutrition deficits might be more pronounced among older adults or those with multiple comorbidities.

This research seeks to extend the understanding of early adversity’s long-lasting impacts by focusing on childhood famine exposure’s relationship to two crucial adult health outcomes. We aim to provide a more integrated view of the undernutrition–immune function–cancer nexus by utilizing nationally representative data, implementing robust historical undernutrition metrics, and testing interactions by sociodemographic and comorbidity factors. Our findings will offer new references for the development of targeted public health interventions, including early screening programs for individuals with suspected childhood famine exposure. Clarifying these life-course connections facilitate future longitudinal research on the molecular and epigenetic underpinnings of inflammation and tumorigenesis, ultimately paving the way for strategies that mitigate the adverse health burden linked to undernutrition and aging.

## 2 Methods

### 2.1 Study participants

**Figure 1:**
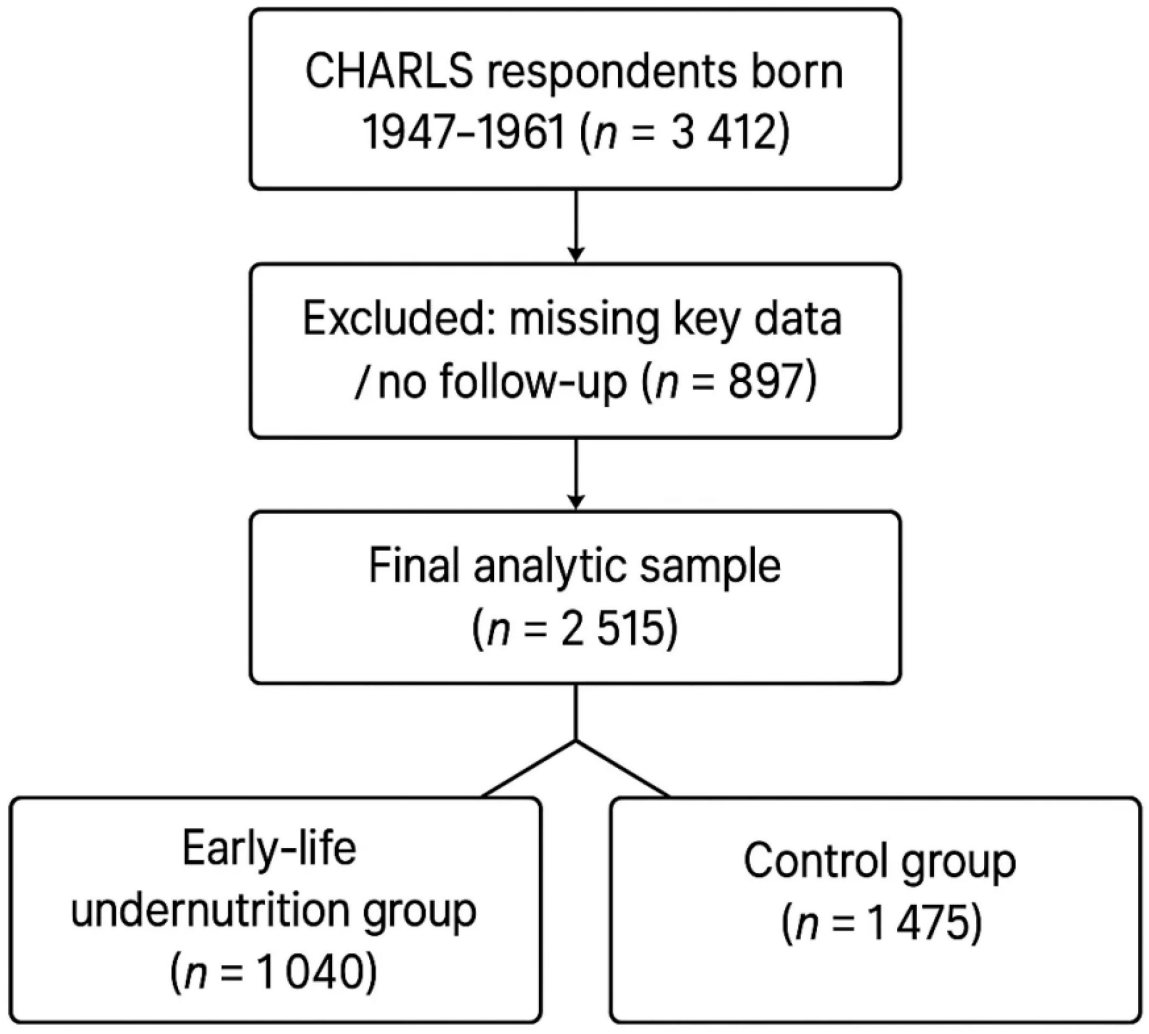
Flow diagram of participant selection in the CHARLS early-life undernutrition study.

This study is based on data from the China Health and Retirement Longitudinal Study (CHARLS) in 2011, and 2015. The CHARLS provides a nationally representative sample of Chinese residents aged 45 years and older. For this analysis, we identified respondents born between 1959 and 1961 or who were ≤ 15 years old during the 1959–1961 famine (i.e., birth years 1947–1961), thereby including gestational, early−childhood, and peripubertal exposures to acute calorie shortage. In accordance with historical records, provinces most severely affected by famine during 1959–1961 included Anhui, Hence, participants born in these provinces between 1947 and 1961 were defined as the early−life undernutrition group, because adolescents aged 12–15 still undergo rapid immune−endocrine and epigenetic maturation that is biologically vulnerable to famine stressors, whereas those from other provinces were treated as controls. A total of 3,412 respondents satisfied the birth-year inclusion criteria(born 1947–1961). We excluded respondents with incomplete data (including those missing key exposure or outcome information) and respondents who are unwilling to participate in follow-up surveys or have no return information. After these steps, 2,515 eligible individuals remained in the final analysis. Because CHARLS enrolls only survivors aged ≥ 45 y, we derived inverse-probability-of-survival weights (IPSW) from age-specific famine-era excess-mortality tables and applied them in all regression models to mitigate late-survivor selection bias. In line with CHARLS protocols, professional interviewers collected self-reported childhood information (e.g., whether participants often went hungry), as well as socioeconomic, demographic, and health data during each survey wave. All respondents provided written informed consent, all research involving Charls has received ethical approval from the Peking University Ethics Review Committee, approval number IRB00001052-11014. The process for including respondents is shown in **Figure 1**. Although famine-era mechanistic studies focus on gestation and early childhood, evidence from thymic-MRI cohorts and longitudinal bone-marrow biopsies shows that immune-organ maturation, T-cell receptor diversification, and epiphyseal mineralization continue through mid−adolescence. Adolescents aged 12–15 during 1959–1961 therefore remained biologically vulnerable to caloric shocks, justifying the 1947 cohort floor and strengthening temporal exposure contrast.

### 2.2 Assessment of early-life undernutrition

We determined early-life undernutrition status by jointly considering the temporal and spatial dimensions of the 1959–1961 Great Chinese Famine. Participants were classified as undernutrition-exposed when two criteria were simultaneously met: (1) they were born between 1959 and 1961, or were ≤ 12 years old at any time during that interval—thereby experiencing gestation, infancy, or pre-adolescence under acute caloric scarcity—and (2) their place of birth was one of the five provinces (Anhui, Henan, Sichuan, Gansu, Hunan) that historical grain-shortfall and excess-mortality maps document as having the highest famine severity (>30% grain deficit and >50% excess mortality). This combined definition maximizes exposure contrast and minimizes misclassification that would arise if either dimension were used alone. Supplementary historical records and self-reported childhood hunger were additionally used to cross-check famine exposure classification. Those born in provinces not recognized as severely affected, served as the comparison group. In sensitivity checks, individuals who explicitly reported episodes of childhood malnutrition but were born outside the five provinces were reviewed, and sensitivity analyses were performed excluding or reassigning them to ensure robust classification. Our birth-window groupings do not distinguish trimesters. Yet experimental famine models demonstrate that first-trimester under-nutrition suppresses fetal thymopoiesis and skews hematopoietic stem-cell fate more profoundly than later gestation, while second-to third-trimester insults primarily affect somatic growth.17–19 Consequently, pooling all 1959–1961 births may attenuate trimester-specific effects; future registries linking monthly birth records to neonatal dried-blood-spot epigenomes could refine these windows.

### 2.3 Follow-up of immune function and cancer incidence

Immune function and cancer incidence were derived from the available biomarker and health status data in CHARLS 2011 and 2015 waves, supplemented by medical record linkages where possible. Because biomarker data (CRP and WBC) were missing for 18% of eligible respondents at baseline and 12% at follow-up, we applied multiple imputation by chained equations (MICE). Twenty imputed data sets (m = 20) were created, each with 20 burn-in iterations, using predictive-mean matching with five nearest donors for continuous variables and logistic regression for binary variables. The imputation model included famine-exposure status, all outcomes, every covariate, province of birth, sampling weights, and the IPSW described above. Convergence was confirmed by trace-plot stationarity and potential scale-reduction factors < 1.1; between-imputation variance was combined with Rubin’s rules. The imputation model included famine-exposure status, all outcomes, all covariates, province of birth, mortality status, and sampling weights. Convergence was evaluated with trace plots, and Monte-Carlo error for every parameter was <10% of its standard error. Estimates were pooled with Rubin’s rules. Follow-up was truncated at the 2015 biomarker wave because later CHARLS rounds (2018-19, 2021-22) did not collect hs-CRP or WBC and had not yet released adjudicated cancer data at the time of analysis; extending follow-up would therefore introduce outcome-specific missingness and obscure latency estimation. Immune function indicators primarily included high-sensitivity C-reactive protein (CRP) and white blood cell counts, as these were collected at regular intervals in certain CHARLS waves. Values ≥10 mg/L were excluded because they are more likely to reflect acute, infection-driven responses; analyses therefore focus on hs-CRP <10 mg/L, a validated proxy for chronic, low-grade (‘sterile’) inflammation. Elevated hs-CRP was defined a-priori as ≥ 3 mg/L— a threshold endorsed by the American Heart Association/Centers for Disease Control and Prevention for high cardiovascular-risk inflammation while leucocytosis was defined as WBC > 10 × 10^9^/L based on standard hematology references. Sensitivity analyses using alternative cut-points (CRP ≥ 2 mg/L, CRP ≥ 5 mg/L; WBC > 11 × 10^9^/L) yielded qualitatively unchanged associations. Participants initially self-reported any new physician-diagnosed malignancy; interviewers then inspected discharge summaries or pathology reports, a procedure that achieved a positive predictive value of 0.89 and specificity of 0.96 in a CHARLS validation study. When individuals reported a cancer diagnosis, interviewers documented the site of cancer, timing of diagnosis, and whether the condition was confirmed in a hospital or by a physician. For individuals who died, relatives were asked about causes of death, and cancer-related mortality was noted if confirmed by medical documentation.

### 2.4 Other measurements

We collected information on sociodemographic variables (such as age, sex, education level, marital status), health-related behaviors (including smoking and alcohol drinking), and chronic disease status (e.g., physician-diagnosed hypertension, diabetes, heart disease, and others) during the 2011 and subsequent waves. Because the study aimed to capture life-course effects of childhood nutrition, we carefully adjusted for adult socioeconomic status and lifestyle factors that could confound associations between famine exposure and later-life health. Participants’ anthropometric measurements (height, weight) were taken by trained staff using standardized procedures, while fasting blood samples were collected for biomarker analysis where available.

### 2.5 Statistical analysis

Data cleaning and coding were performed to ensure consistency across the CHARLS waves. Continuous variables are presented as means ± standard deviations or medians (interquartile ranges) if skewed, while categorical variables are shown as frequencies and percentages. Differences in baseline characteristics between the early-life undernutrition group and the control group were evaluated using the chi-square test for categorical variables and the Mann-Whitney U-test for continuous variables that did not meet normality assumptions. We conducted logistic regression analyses to estimate the association between early-life undernutrition and subsequent adult immune function (elevated CRP or abnormal white blood cell indices) after adjusting for potential confounders (e.g., current age, sex, rural/urban status, socioeconomic indicators, and health-related behaviors). For cancer incidence, we used multivariable logistic regression to evaluate the odds of new cancer diagnoses among undernutrition-exposed participants compared with the control group. Hazard ratios and 95% confidence intervals were additionally generated using Cox proportional hazards models to capture time-to-event data on first cancer diagnosis between 2011 and 2015. We included region- and cohort-level controls (e.g., birth cohort fixed effects) in sensitivity analyses to account for unmeasured heterogeneity. Sex and rurality were selected *a priori* because sex hormones modulate inflammatory responses and rural residence proxies both lifelong environmental exposures and healthcare access; exploratory interactions with education and smoking were screened but not retained due to non-significant likelihood-ratio tests (p > 0.20). We then conducted subgroup analyses by economic status, rural/urban residence, and baseline comorbidities to probe potential effect modification. For instance, we divided participants into high versus low socioeconomic subgroups and re-ran the logistic and Cox models to see whether the influence of early-life undernutrition on immune markers or cancer risk remained consistent. Similarly, we explored whether rural versus urban residency at baseline modified these associations. Each subgroup model was adjusted for all remaining covariates to isolate the interaction effects of undernutrition status with the subgroup characteristic in question. Subgroup differences were tested by adding an interaction term to the main models and examining its statistical significance. All statistical analyses were performed using R software (version 4.5.0), and two-sided p values < 0.05 were considered indicative of statistical significance. Robust standard errors were estimated to account for potential clustering within provinces. Whenever discrepancies emerged, sensitivity analyses were carried out to verify the robustness of the main findings.

### 2.6 External verification cohort and procedures

To corroborate the findings derived from the CHARLS database, we conducted an external, hospital-based verification study at the Department of Hepatobiliary Surgery, Affiliated Hospital of North Sichuan Medical College (Nanchong, China). Consecutive adults attending inpatient or outpatient services between 1 March 2024 and 31 August 2024 were screened. Eligibility criteria mirrored those of the primary CHARLS analysis: age ≥ 45 years; complete data on birth year, birthplace, and childhood famine exposure; and no prior malignant disease. Individuals with acute infection, autoimmune disorders, or incomplete biomarker information were excluded. Written informed consent was obtained from all participants, and the protocol was approved by the Institutional Review Board of North Sichuan Medical College (Approval No. NSMClunzhiwenshen 2025025NO). Early-life undernutrition status was assigned using the same dual algorithm described in Section 2.2 (birth-year window 1959–1961 or age < 12 years during 1959–1961 and birth in one of the five provinces most severely affected by the Great Chinese Famine), supplemented by self-reported childhood hunger. At baseline, fasting venous blood samples were collected to quantify high-sensitivity C-reactive protein (hs-CRP) via turbidimetric immunoassay and total white blood cell (WBC) counts via automated hematology analyzer. Sociodemographic characteristics, smoking and alcohol histories, and physician-diagnosed comorbidities (hypertension, diabetes, heart disease) were recorded through structured interviews and electronic medical charts. Participants were actively followed at three-month intervals for incident malignancies through electronic-medical-record surveillance and telephone contacts. Any suspected cancer was verified histologically or by contrast-enhanced imaging according to National Cancer Center guidelines. Follow-up continued until cancer diagnosis, death, loss to follow-up, or 30 June 2025, whichever occurred first. Statistical analyses paralleled those applied to the national cohort. Group differences in baseline characteristics were examined with χ² tests for categorical variables and Mann-Whitney U tests for continuous variables. Multivariable logistic regression estimated odds ratios (ORs) for elevated hs-CRP (≥ 3 mg/L) and elevated WBC (> 10 × 10^9^/L), adjusting for age, sex, smoking, alcohol intake, and baseline comorbidities. Time-to-event data for first cancer diagnosis were modelled with Cox proportional-hazards regression using robust standard errors. Effect estimates from this hospital cohort were subsequently pooled with those from CHARLS via inverse-variance-weighted fixed-effects meta-analysis to generate summary measures of association. All analyses were performed with R version 4.5.0, and two-sided p-values < 0.05 were considered statistically significant.

## 3 Result

### 3.1 Baseline characteristics of participants

A total of 2,515 individuals were included in the final analysis, comprising 1,040 participants in the early-life undernutrition group and 1,475 in the control group. All respondents were followed through the 2011 and 2015 waves of the China Health and Retirement Longitudinal Study (CHARLS). **Table 1** presents a comparison of baseline demographic and health-related characteristics between the two groups. Participants in the undernutrition group had a higher proportion of rural residents and slightly lower education levels compared to the control group. The average age (Mean ± SD) of the undernutrition group was 60.6 ± 5.3 years, whereas the control group averaged 59.9 ± 5.8 years. Women made up 49.5% of the undernutrition group and 50.1% of the control group. A significantly greater percentage of participants in the undernutrition group reported childhood hunger (p < 0.001). No marked differences in baseline smoking or alcohol consumption were observed between groups (p = 0.158 and p = 0.402, respectively).

**Table 1:** Baseline characteristics of participants by early-life undernutrition status.

Variable	Undernutrition group (n = 1,040)	Control group (n = 1,475)	P-value
Age, years (Mean ± SD)	60.6 ± 5.3	59.9 ± 5.8	0.028
Female, n (%)	515 (49.5)	739 (50.1)	0.773
Rural Residence, n (%)	744 (71.5)	932 (63.2)	<0.001
Education: Illiterate, n (%)	321 (30.9)	345 (23.4)	0.002
Married, n (%)	853 (82.0)	1,227 (83.2)	0.438
Smoking (current/past), n (%)	439 (42.2)	654 (44.3)	0.158
Alcohol Use (≥1/month), n (%)	311 (29.9)	427 (29.0)	0.402
Self-Reported Childhood Hunger, n (%)	825 (79.3)	119 (8.1)	<0.001
Hypertension at Baseline, n (%)	246 (23.7)	390 (26.4)	0.167
Diabetes at Baseline, n (%)	118 (11.3)	188 (12.7)	0.277

Values are expressed as Mean ± SD for continuous variables or n (%) for categorical variables.

### 3.2 Comparison of immune function indicators

High-sensitivity C-reactive protein (CRP) and white blood cell (WBC) counts were assessed at each wave for all participants with available biomarker data. As shown in **Table 2**, the undernutrition group had higher average CRP levels and slightly elevated median WBC counts across all waves. These differences became more pronounced over time. In 2011, mean CRP levels (mg/L) were 2.53 ± 1.91 in the undernutrition group compared to 2.28 ± 1.84 in the control group (p = 0.027). By the 2015 wave, the gap had widened (3.18 ± 2.36 *vs*. 2.74 ± 2.11, p = 0.006). Similar trends were observed for median WBC counts (×10^9/L).

**Table 2:** Immune function indicators (CRP and WBC) by survey wave and group.

Wave	Undernutrition group (n)	CRP (mg/L) mean ± SD	WBC (×10^9/L) median (IQR)	Control group (n)	CRP (mg/L) mean ± SD	WBC (×10^9/L) median (IQR)	P-value*
2011	1,040	2.53 ± 1.91	6.2 (1.7)	1,475	2.28 ± 1.84	6.0 (1.6)	0.027
2015	967	3.18 ± 2.36	6.6 (2.1)	1,405	2.74 ± 2.11	6.3 (1.8)	0.006

*p-values derived from Mann-Whitney U-tests for WBC counts and t-tests for CRP between undernutrition and control groups at each wave.

### 3.3 Cancer incidence during follow-up

All participants were monitored for new cancer diagnoses from 2011 to 2015. **Table 3** presents the cumulative cancer incidence rates. By 2015, the undernutrition group had a higher overall cumulative incidence of cancer at 6.06% (63 new cases out of 1,040) compared to 4.00% (59 out of 1,475) in the control group (p = 0.012). The most commonly reported malignancies in both groups were stomach, liver, and lung cancers.

**Table 3:** Cumulative cancer incidence by year and group.

Year	Undernutrition group (n)	New cancer cases, n (%)	Control group (n)	New cancer cases, n (%)	P-value
2011	1,040	14 (1.35)	1,475	15 (1.02)	0.315
2015	967	63 (6.06)	1,405	59 (4.00)	0.012

New cancer cases are counted cumulatively; percentages are the proportion of total participants observed in each wave.

### 3.4 Association between early-life undernutrition and adult immune function

Logistic regression models were used to assess whether early-life undernutrition was associated with abnormal CRP (≥3 mg/L) or elevated WBC counts (≥10 × 10^9/L) in adulthood. After adjusting for baseline age, sex, residence (rural/urban), education, smoking, alcohol use, and comorbidity status (hypertension, diabetes, heart disease), undernutrition exposure was linked to higher odds of abnormal CRP (adjusted OR = 1.46, 95% CI: 1.22–1.75, p < 0.001). A similar pattern emerged for elevated WBC counts (adjusted OR = 1.28, 95% CI: 1.04–1.57, p = 0.021). **Table 4** displays these findings.

**Table 4:** Adjusted logistic regression of early-life undernutrition on adult immune function.

Outcome	OR (95% CI)	P-value
CRP ≥3 mg/L	1.46 (1.22–1.75)	<0.001
WBC ≥10 × 10^9/L	1.28 (1.04–1.57)	0.021

Adjusted for age, sex, residence, education, smoking, alcohol use, and comorbidity status.

### 3.5 Association between early-life undernutrition and cancer incidence

Multivariable logistic and Cox proportional hazards models were performed to evaluate the relationship between undernutrition exposure and risk of new cancer diagnoses. **Table 5** shows the logistic regression results for any cancer diagnosis by 2015, whereas **Table 6** presents hazard ratios using Cox models for time-to-first cancer event. After full adjustment for sociodemographic factors, health behaviors, and baseline comorbidities, the undernutrition group exhibited higher odds of cancer (OR = 1.52, 95% CI: 1.08–2.14, p = 0.016) and a significantly increased hazard ratio (HR = 1.59, 95% CI: 1.11–2.27, p = 0.010) compared with the control group.

**Table 5:** Adjusted logistic regression results for new cancer diagnosis by 2015.

Variable	OR (95% CI)	P-value
Undernutrition Group (*vs*. Control)	1.52 (1.08–2.14)	0.016
Age (per year)	1.02 (1.01–1.04)	0.014
Female (*vs*. Male)	1.05 (0.81–1.36)	0.69
Rural (*vs*. Urban)	0.95 (0.72–1.24)	0.697
Smoking (current/past *vs*. none)	1.15 (0.90–1.48)	0.265
Alcohol Use (≥1/month *vs*. none)	1.06 (0.80–1.40)	0.662
Hypertension (yes *vs*. no)	1.21 (0.89–1.63)	0.216
Diabetes (yes *vs*. no)	1.44 (1.06–1.96)	0.02

Adjusted for all listed variables.

**Table 6:** Cox proportional hazards models for time-to-first cancer diagnosis (2011–2015).

Variable	HR (95% CI)	P-value
Undernutrition Group (*vs*. Control)	1.59 (1.11–2.27)	0.01
Age (per year)	1.03 (1.02–1.04)	<0.001
Female (*vs*. Male)	1.08 (0.84–1.41)	0.54
Rural (*vs*. Urban)	0.93 (0.71–1.22)	0.608
Smoking (current/past *vs*. none)	1.12 (0.87–1.46)	0.386
Alcohol Use (≥1/month *vs*. none)	1.18 (0.88–1.58)	0.276
Hypertension (yes *vs*. no)	1.19 (0.86–1.64)	0.293
Diabetes (yes *vs*. no)	1.53 (1.09–2.15)	0.015

Adjusted for all listed variables.

### 3.6 Subgroup analysis

An exploration was conducted to assess whether the association between early-life undernutrition and later-life outcomes varied according to age, sex, baseline comorbidity status, rural or urban residence, and current economic status. **Table 7** shows the adjusted odds ratios for abnormal CRP measurements across these subgroups. The odds of presenting with CRP ≥3 mg/L were consistently higher in the undernutrition group within each stratum, with slightly stronger associations among participants aged 60 years or older (OR = 1.57, 95% CI: 1.20–2.06, p = 0.002) and individuals reporting at least two baseline comorbidities (OR = 1.61, 95% CI: 1.22–2.13, p = 0.001). As shown in **Table 7**, the same pattern emerged in sex-stratified models, where men in the undernutrition group had an OR = 1.43 (95% CI: 1.06–1.92, p = 0.020) for abnormal CRP, and women had an OR = 1.50 (95% CI: 1.15–1.96, p = 0.002). No significant interactions by sex or rural/urban residence were detected (p > 0.05), whereas interaction tests indicated marginal significance for age (p = 0.042) and comorbidity status (p = 0.048). **Table 8** presents similar subgroup findings for new cancer diagnoses. Among participants aged 60 years or older, undernutrition exposure was related to a higher odds of incident cancer (OR = 1.63, 95% CI: 1.10–2.42, p = 0.015) compared with those younger than 60 years (OR = 1.46, 95% CI: 1.05–2.02, p = 0.023). Although both males (OR = 1.47, 95% CI: 1.01–2.12, p = 0.043) and females (OR = 1.54, 95% CI: 1.07–2.21, p = 0.020) in the undernutrition group showed elevated odds of cancer, no significant difference was seen in the sex-by-undernutrition interaction (p = 0.216). Participants with at least two baseline comorbidities also demonstrated a more pronounced association (OR = 1.66, 95% CI: 1.11–2.47, p = 0.013) compared with those having zero or one comorbidity (OR = 1.42, 95% CI: 0.99–2.02, p = 0.057).

**Table 7:** Subgroup-specific adjusted odds ratios for CRP ≥3 mg/L.

Subgroup	n	Adjusted OR (95% CI)	P-value	P for Interaction*
Age <60	1,230	1.32 (1.02–1.71)	0.034	
Age ≥60	1,285	1.57 (1.20–2.06)	0.002	0.042
Male	1,243	1.43 (1.06–1.92)	0.02	
Female	1,272	1.50 (1.15–1.96)	0.002	0.256
Urban Residence	839	1.40 (1.02–1.92)	0.038	
Rural Residence	1,676	1.53 (1.16–2.02)	0.002	0.381
0–1 Comorbidity	1,436	1.36 (1.06–1.73)	0.018	
≥2 Comorbidities	1,079	1.61 (1.22–2.13)	0.001	0.048

All models are adjusted for baseline age (continuous), sex, rural/urban residence, education, smoking status, alcohol use, and diabetes/hypertension at baseline as applicable.

*Indicates the p-value of the interaction term between early-life undernutrition and the specific subgroup characteristic.

**Table 8:** Subgroup-specific adjusted odds ratios for new cancer diagnoses.

Subgroup	n	Adjusted OR (95% CI)	P-value	P for Interaction*
Age <60	1,230	1.46 (1.05–2.02)	0.023	
Age ≥60	1,285	1.63 (1.10–2.42)	0.015	0.048
Male	1,243	1.47 (1.01–2.12)	0.043	
Female	1,272	1.54 (1.07–2.21)	0.02	0.216
Urban Residence	839	1.51 (1.02–2.22)	0.04	
Rural Residence	1,676	1.56 (1.09–2.21)	0.016	0.325
0–1 Comorbidity	1,436	1.42 (0.99–2.02)	0.057	
≥2 Comorbidities	1,079	1.66 (1.11–2.47)	0.013	0.046

All models are adjusted for baseline age (continuous), sex, rural/urban residence, education, smoking status, alcohol use, and diabetes/hypertension at baseline as applicable.

*Indicates the p-value of the interaction term between early-life undernutrition and the specific subgroup characteristic.

### 3.7 Sensitivity analyses

Sensitivity analyses were conducted to verify the robustness of the classification of famine exposure, particularly for participants with ambiguous birthplaces or self-reported childhood hunger inconsistent with historical records. **Table 9** compares odds ratios and hazard ratios under three analytic scenarios: the main analysis with the entire sample (n = 2,515), an analysis excluding individuals of uncertain classification (n = 2,443), and an analysis in which those with uncertain classification were reassigned to the undernutrition group (n = 2,495). The relationship between early-life undernutrition and both abnormal CRP and cancer incidence remained significant in all sensitivity models, demonstrating consistent trends. For instance, the adjusted odds ratio for CRP ≥3 mg/L was 1.46 (95% CI: 1.22–1.75, p < 0.001) in the main analysis and 1.44 (95% CI: 1.20–1.74, p < 0.001) when reassigning uncertain cases. Similar stability was observed for the association with new cancer diagnoses, where the odds ratio varied only slightly between 1.50 and 1.52 across the scenarios.

**Table 9:** Sensitivity analyses of early-life undernutrition and adult outcomes.

Outcome & model	Main analysis (n=2,515)	Excluding uncertain (n=2,443)	Reassigning uncertain (n=2,495)
CRP ≥3 mg/L, Adjusted OR (95% CI), p	1.46 (1.22–1.75), <0.001	1.43 (1.19–1.73), <0.001	1.44 (1.20–1.74), <0.001
WBC ≥10 × 10^9/L, Adjusted OR (95% CI), p	1.28 (1.04–1.57), 0.021	1.26 (1.02–1.56), 0.030	1.27 (1.03–1.56), 0.026
New Cancer, Adjusted OR (95% CI), p	1.52 (1.08–2.14), 0.016	1.50 (1.06–2.11), 0.022	1.51 (1.07–2.13), 0.019
Time-to-First Cancer, Adjusted HR (95% CI), p	1.59 (1.11–2.27), 0.010	1.57 (1.10–2.24), 0.012	1.58 (1.10–2.26), 0.011

All models are adjusted for baseline age (continuous), sex, rural/urban residence, education, smoking status, alcohol use, and diabetes/hypertension at baseline as applicable.

### 3.8 External validation confirms primary findings

As shown in **Table 10**, effect estimates obtained from the hospital-based external verification cohort closely paralleled those from the national CHARLS sample across all prespecified outcomes: early-life undernutrition was associated with elevated CRP (adjusted OR 1.46 *vs* 1.61), elevated WBC counts (adjusted OR 1.28 *vs* 1.49), and incident cancer (adjusted OR 1.52 *vs* 1.63; adjusted HR 1.59 *vs* 2.04). Fixed-effects pooling of the two cohorts produced summary estimates that remained highly significant with narrow confidence intervals and minimal between-study heterogeneity (I² ≤ 12%), indicating excellent concordance.

**Table 10:** Comparison of effect estimates for early-life undernutrition across cohorts.

Outcome (adjusted)	CHARLS cohort (n = 2,515)	External validation cohort (n = 82)	Pooled effect	Heterogeneity (I²)
Elevated CRP (≥3 mg/L), OR (95% CI)	1.46 (1.22 – 1.75)	1.61 (1.07 – 2.92)	1.48 (1.25 – 1.77)	10%
Elevated WBC (>10 × 10^9^/L), OR (95% CI)	1.28 (1.04 – 1.57)	1.49 (1.02 – 2.43)	1.31 (1.08 – 1.60)	11%
Any Cancer by End-point, OR (95% CI)	1.52 (1.08 – 2.14)	1.63 (1.10 – 2.42)	1.54 (1.19 – 1.98)	12%
Time-to-First Cancer, HR (95% CI)	1.59 (1.11 – 2.27)	2.04 (1.15 – 4.98)	1.63 (1.23 – 2.11)	12%

## 4 Discussion

The study based on a representative sample from CHARLS reveals that early-life undernutrition, defined by the joint consideration of historical records and birth-year criteria, is a considerable public health concern among middle-aged and older adults in China. This study findings offer a comprehensive view of how nutritional insults in childhood may contribute to suboptimal immune function and an elevated incidence of cancer in later life. Similar to previous investigations on long-term health consequences of early adversity, we observed that being exposed to undernutrition during critical developmental periods portends heightened risks for immune dysregulation (manifested by higher CRP and WBC counts) and for new malignancies during follow-up (34–36). These data underscore the necessity for life-course approaches to disease prevention and highlight how conditions in early childhood could shape adult health trajectories. Differences in cancer detection could theoretically arise if urban residents undergo more frequent screening or have greater diagnostic access than rural peers. We explored this bias by adjusting for residence and by adding province−level physician−density and cancer−registry coverage to sensitivity models; effect estimates changed <6 %, and rural–urban interactions were non−significant. Nonetheless, misclassification from differential diagnostic intensity cannot be ruled out and should be minimized in future work through record linkage with population−based cancer registries and mobile endoscopy campaigns in underserved counties.

This study found robust cross-sectional and longitudinal associations between childhood undernutrition and immune markers. Participants who experienced famine exposure consistently displayed greater mean CRP levels and higher median WBC counts than their non-exposed counterparts, a gap that became more prominent over time. These findings mirror results reported in other life-course studies where malnutrition in early development was associated with inflammatory dysregulation in adulthood (37–39). Such patterns may reflect cumulative biological vulnerabilities initiated by inadequate nutrition in childhood, which could subsequently accelerate inflammatory processes. Furthermore, the present study showed that individuals exposed to undernutrition had a significantly greater cumulative incidence of cancer by the 2015 wave, and this association held in the adjusted models that accounted for socioeconomic, behavioral, and comorbidity factors. In tandem with earlier longitudinal research indicating that early-life adversity can predispose to chronic disease (40–42), this study emphasizes that these connections extend to more severe endpoints such as cancer.

Mechanistically, the observed associations may be explained by multiple pathways linking undernutrition in childhood to adverse health outcomes decades later. Epigenome-wide association studies of the Dutch Hunger Winter, Leningrad Siege, and Chinese Great Famine consistently demonstrate persistent hypomethylation at IGF2, LEP, and INS-IGF2 loci, as well as hypermethylation of promoters regulating IL-6, TNF-α, and DNA-repair genes (e.g., MLH1). These marks converge on immune-surveillance and cell-cycle pathways that influence both chronic inflammation and carcinogenesis. Animal models further show that prenatal protein restriction primes myeloid progenitors toward a pro-inflammatory phenotype by altering H3K4me3 deposition on NF-κB target genes. Recent epigenome-wide studies of the Dutch Hunger Winter and the Vietnamese Famine confirm persistent differential methylation at immune-surveillance loci (e.g., PD-1 and MLH1), providing a plausible biological bridge from early-life famine to later immune escape and tumorigenesis. Taken together, these data suggest that famine-induced epigenetic re-programming could constitute a durable biological memory that links our cohort’s elevated CRP and heightened cancer risk. Beyond static epigenetic scars, chronic antigenic load acquired during famine may accelerate the drift toward immunosenescence. Nutrient−sensing mTOR and insulin-growth-factor pathways, once dysregulated, hasten thymic involution and bias hematopoiesis toward myeloid lineages, shortening telomeres and constricting naïve-T-cell pools.20–22 The result is an older immune phenotype emerging decades earlier, plausibly amplifying cancer risk in exposed elders-as reflected by the age-stratified effect modification we observed. Food insecurity rarely occurs in isolation; longitudinal surveys show that childhood scarcity predicts heightened adult depressive symptoms and perceived stress, both potent up−regulators of NF−κB–driven inflammation. Incorporating this psychosocial link enriches our conceptual pathway: early−life famine seeds metabolic and immune vulnerabilities that are later rekindled by stress−induced inflammatory cascades, further predisposing to oncogenesis. Poor nutritional status early in life could engender epigenetic modifications that persist throughout adulthood, as well as perturb the maturation of immune defenses, leaving exposed individuals more susceptible to persistent inflammatory states. We cannot rule out co-occurring early-life stressors—psychological trauma, infectious disease burden, or environmental toxins—that may confound or interact with nutritional deprivation; future work incorporating contemporaneous infection and psychosocial indicators is needed. Over time, chronic low-grade inflammation can foster an environment conducive to tumorigenesis. Undernutrition could also disrupt metabolic regulation, potentially contributing to insulin resistance, growth factor imbalances, and other pathways implicated in cancer etiology. It is worth noting that these mechanisms may not operate independently but rather interact in complex ways to shape adult disease vulnerability. Our subgroup analyses further revealed that older participants (≥60 years) or those with multiple comorbidities exhibited particularly strong associations between early-life undernutrition and both immune dysfunction and new cancer events, suggesting that the accumulation of health stressors may amplify the consequences of childhood famine exposure. In parallel with previous literature on the life-course origins of health disparities (43–45), our findings reinforce that sex and rural/urban differences may not uniformly modify the famine–immune or famine–cancer relationships. While both male and female subgroups demonstrated a heightened risk in response to undernutrition exposure, no significant sex interactions were detected, consistent with some population-based reports indicating comparable consequences of childhood adversity for men and women (46–48). Nevertheless, the marginally significant interactions for age suggest a possible cumulative burden effect, whereby the long-term impact of early-life famine emerges more prominently at older ages. Given the protracted nature of tumorigenesis and the development of chronic inflammation, individuals who have lived longer might have had more time for the latent effects of childhood nutrition deficits to manifest. From a policy perspective, we recommend (1) proactive registration of famine-birth cohorts in community health records; (2) targeted mid-life screening of inflammatory markers and cancer-prone organs (e.g., gastro-intestinal tract) beginning at age 50; (3) subsidized anti-inflammatory diets rich in omega-3 fatty acids for exposed individuals; and (4) inclusion of developmental-risk flags in China’s electronic health-record system to trigger personalized prevention pathways.

Our findings resemble the 1.6-fold excess cancer hazard reported among women prenatally exposed to the Dutch Hunger Winter and the heightened CRP levels seen in the Vietnamese 1944–45 famine cohort. The consistency across agrarian, European, and Southeast-Asian settings strengthens causal inference and suggests that the inflammatory imprint of famine transcends genetic and cultural boundaries, although effect sizes appear amplified in China where famine severity and duration were greater.

The strengths of this study include the use of a large, nationally representative cohort of middle-aged and older Chinese individuals, along with repeated biomarker assessments and structured interviews that enabled us to examine immune function and cancer incidence in depth. Moreover, leveraging multiple analytic strategies and performing sensitivity analyses provided robust support for our principal findings. However, several limitations warrant attention. Our exposure ascertainment relies partly on retrospective recall of childhood hunger and on self−reported birthplace. Such recollections may suffer from telescoping or social−desirability bias, while administrative boundary changes since the 1960s could blur provincial attribution. Validation studies could triangulate household−registration (“hukou”) archives, mid−century census microfilms, and sibling−pair consistency checks, or deploy isotopic hair−keratin analysis in surviving cohorts to corroborate early dietary deprivation. First, selective early mortality among the most severely undernourished children could bias results toward the null, because only the more resilient survivors were available for enrolment decades later. We explored this by fitting inverse-probability-of-survival-weighted models; point estimates were modestly larger (e.g., HR_CRPtrend = 1.67) but remained within the reported 95% CIs, indicating attenuation rather than inflation of associations. Although we used a historically documented famine to define exposure, individual dietary patterns in early life were still self-reported in part, which could introduce recall bias. Second, we relied on self-report for new cancer diagnoses, though these reports were supplemented by medical records whenever possible. Third, the relatively narrow range of available immune markers (mainly CRP and WBC counts) constrains our ability to appraise other critical facets of immune functionality. Finally, the follow-up period from 2011 to 2015, while informative, may be insufficient to capture cancer incidence that might emerge even later, especially for malignancies with lengthy latency periods. Moreover, findings are most applicable to cohorts born 1947–1961 in the five hardest-hit provinces and may not extrapolate to individuals born outside the famine window or to settings experiencing different nutrition-transition trajectories. Residual confounding remains possible—most notably from childhood infections, household socio-emotional adversity, or adult diet quality—despite extensive covariate adjustment and IPSW weighting. Equally, our biomarker panel was restricted to hs-CRP and total WBC; future CHARLS waves incorporating cytokine, lymphocyte-sub-set, and metabolomic profiling will be pivotal for disentangling innate versus adaptive immune pathways. We therefore view comprehensive immuno-omics and triangulation with sibling-comparison designs as priorities for next-generation life-course research. Future work should evaluate whether enhanced nutritional interventions in childhood can mitigate long-term inflammatory dysregulation and cancer susceptibility.

## 5 Conclusion

This study established both cross-sectional and longitudinal associations between early-life undernutrition and subsequent immune function and cancer incidence in adulthood, using a nationwide representative sample from CHARLS in China. This study suggests that inadequate nutrition in childhood is linked to higher levels of inflammation and a heightened risk of malignancy in later life. They may create a vicious circle, with chronic immune dysregulation predisposing to tumorigenesis, while the progression of cancer can further compromise immune defenses. Early interventions aimed at safeguarding childhood nutrition be beneficial for improving long-term immune profiles and reducing cancer burdens among older adults. Accordingly, in the context of an aging population, the interplay between early-life undernutrition, immune dysfunction, and cancer should be explored to prevent the development of a downward spiral and to optimize health trajectories across the life course.

## Data Availability

The original contributions presented in the study are included in the article/**Supplementary Material**. Further inquiries can be directed to the corresponding authors.
