# Supplementary material for: Early-life undernutrition, immune dysregulation, and cancer incidence in later life: a national life-course analysis from the China health and retirement longitudinal study
Source: Front Immunol. 2025 Aug 27;16:1602290. doi: 10.3389/fimmu.2025.1602290 (PMC12420247; doi:10.3389/fimmu.2025.1602290)
Supplement: Supplementary file 1 [file Table1.docx]

**Table S1. Province-Level Classification of Famine Severity and Birth-Year Criteria**

| **Province Group** | **Included Provinces** | **Birth Years Considered for Famine Exposure** | **Definition/Notes** |
| --- | --- | --- | --- |
| Severely Affected (Undernutrition Group) | Anhui, Henan, Sichuan, Gansu, Hunan | 1947–1961 (with special emphasis on 1959–1961) | Historical records show these five provinces had the highest famine severity. Individuals born 1959–1961 or under age 12 at any point during 1959–1961 in these provinces were classified as “hungry.” |
| Comparison (Control Group) | All other provinces | 1947–1961 (with the same birth-year range) | Participants from provinces not recognized as severely affected by the 1959–1961 famine were used as controls. |
| Ambiguous Classification (Sensitivity Checks) | - | 1947–1961 (but conflicting or missing birthplace/hunger data) | Participants who self-reported childhood undernutrition but were born outside the above five provinces, or had incomplete/contradictory records. Handled in sensitivity analyses. |

**Table S2. CRP and WBC Cut-offs Used in the Analysis**

| **Marker** | **Normal Range** | **Elevated Range** | **Reference / Rationale** |
| --- | --- | --- | --- |
| C-Reactive Protein | <3 mg/L | ≥3 mg/L | Common threshold for low-grade inflammation |
| White Blood Cell Count | 4–10 × 10^9/L | >10 × 10^9/L | Standard clinical reference range |

**Table S3. Variance Inflation Factors (VIF) in Logistic Regression Models**

| **Covariate** | **Elevated CRP Model VIF** | **Elevated WBC Model VIF** | **Cancer Incidence Model VIF** |
| --- | --- | --- | --- |
| Age (continuous) | 1.22 | 1.28 | 1.31 |
| Sex | 1.36 | 1.34 | 1.41 |
| Rural Residence (vs. Urban) | 1.14 | 1.17 | 1.09 |
| Education (categorical) | 1.18 | 1.22 | 1.25 |
| Smoking Status (yes/no) | 1.09 | 1.15 | 1.16 |
| Alcohol Use (≥1/month vs. no) | 1.11 | 1.1 | 1.13 |
| Hypertension (yes/no) | 1.06 | 1.07 | 1.08 |
| Diabetes (yes/no) | 1.05 | 1.09 | 1.19 |

**Table S4. Hosmer and Lemeshow Goodness-of-Fit Tests for Logistic Regression Models**

| **Model** | **Chi-squared** | **df** | **p-value** |
| --- | --- | --- | --- |
| Elevated CRP (≥3 mg/L), Overall | 7.28 | 8 | 0.506 |
| Elevated WBC (≥10 × 10^9/L), Overall | 5.91 | 8 | 0.657 |
| New Cancer Incidence (2011–2015), Overall | 9.62 | 8 | 0.292 |
| CRP Model, Subgroup: Age ≥60 | 6.31 | 8 | 0.613 |
| CRP Model, Subgroup: Female | 8.02 | 8 | 0.431 |
| Cancer Model, Subgroup: Rural Residence | 7.76 | 8 | 0.458 |
| Cancer Model, Subgroup: ≥2 Comorbidities | 4.85 | 8 | 0.773 |

**Table S5. Sensitivity Analysis Scenarios for Famine-Exposure Classification**

| **Scenario** | **Description** | **Sample Size (n)** | **Key Finding** |
| --- | --- | --- | --- |
| **Main Analysis** | Used the strict province-based criterion + birth-year range (1947–1961). Ambiguous cases included (n=72), but coded as “control.” | 2,515 | Undernutrition associated with significantly higher risk of elevated CRP (OR=1.46, p<0.001) and new cancer (OR=1.52, p=0.016). |
| **Excluding Ambiguous** | Completely removed participants who self-reported hunger but were born outside the 5 famine provinces (n=72). | 2,443 | Results remained stable (CRP OR=1.43, p<0.001; new cancer OR=1.50, p=0.022). |
| **Reassigning Ambiguous to Undernutrition** | Classified ambiguous participants as “hungry” if they reported childhood malnutrition or prolonged hunger (n=68 of 72). | 2,495 | Findings persisted (CRP OR=1.44, p<0.001; new cancer OR=1.51, p=0.019), confirming robustness to classification changes. |

**Table S6. Detailed Breakdown of New Cancer Diagnoses (2011–2015)**

| **Cancer Type** | **Undernutrition Group** (n=1,040) | **Control Group** (n=1,475) | **p-value** |
| --- | --- | --- | --- |
| Stomach | 16 (1.54%) | 15 (1.02%) | 0.211 |
| Liver | 11 (1.06%) | 10 (0.68%) | 0.346 |
| Lung | 10 (0.96%) | 8 (0.54%) | 0.182 |
| Colorectal | 9 (0.87%) | 9 (0.61%) | 0.48 |
| Other Cancers | 17 (1.63%) | 17 (1.15%) | 0.241 |
| **Total New Cancers** | **63 (6.06%)** | **59 (4.00%)** | **0.012** |
